# Psychedelics and the Serotonin Hypothesis of Eating Disorders

**DOI:** 10.3390/brainsci15080893

**Published:** 2025-08-21

**Authors:** Dean Bilenker, Nicole M. Avena

**Affiliations:** 1 SunCloud Health, Chicago, IL 60614, USA; 2 Department of Neuroscience, Icahn School of Medicine at Mount Sinai, New York, NY 10021, USA; 3 Department of Psychology, Princeton University, Princeton, NJ 08540, USA

**Keywords:** eating disorder, psychedelic therapy, serotonin, 5-HT, mental health, psychiatry

## Abstract

Recent advances in psychedelic research have renewed interest in their therapeutic potential for psychiatric disorders characterized by cognitive and behavioral rigidity. This review examines the rationale for using serotonergic psychedelics—particularly 5-HT_2_A receptor agonists such as psilocybin—in the treatment of eating disorders (EDs), including anorexia nervosa (AN), bulimia nervosa (BN), and binge eating disorder (BED). The paper contextualizes these interventions within the broader serotonin hypothesis of EDs, emphasizing serotonergic dysregulation and impaired cognitive flexibility as central features of these conditions. Drawing from animal models, human neuroimaging studies, and emerging clinical trials, the authors outline how psychedelics may promote neuroplasticity and psychological insight through modulation of 5-HT_2_A signaling. Preliminary evidence from open-label studies suggests psilocybin may improve ED symptoms and quality of life, though findings are early and methodologically limited. The paper also reviews data on ayahuasca, MDMA, and non-psychedelic serotonergic agents, highlighting both the promise and complexity of psychedelic-assisted therapy in EDs. The authors conclude that while further controlled trials are needed to clarify efficacy, safety, and optimal treatment parameters, psychedelics offer a novel, mechanistically distinct avenue for addressing entrenched ED psychopathology.

Psychedelics are a class of psychoactive compounds that induce profound but transient alterations in perception, mood, and cognition. These effects are typically mediated by agonism at the serotonin 2A receptor (5-HT_2_A), especially in cortical regions involved in introspection, emotion, and sensory integration [1]. So-called “classical psychedelics” such as lysergic acid diethylamide (LSD), psilocybin (found in certain mushrooms), mescaline (from peyote), and dimethyltryptamine (DMT)—share this mechanism and are structurally related to serotonin. By contrast, ketamine, although sometimes grouped with psychedelics for its dissociative and antidepressant properties, operates primarily through NMDA receptor antagonism and is thus pharmacologically distinct [2]. 3,4-Methylenedioxymethamphetamine (MDMA, “ecstasy”) exerts its primary effects by reversing the serotonin transporter (SERT), leading to a surge of extracellular serotonin along with secondary increases in dopamine and norepinephrine [3]. Unlike classical psychedelics, MDMA produces minimal perceptual distortion and is better understood as a hybrid stimulant–entactogen.

Interest in the therapeutic potential of psychedelics surged in the mid-20th century, with early studies exploring their use in alcoholism, depression, and existential distress in terminal illnesses [4]. This research was largely halted by regulatory crackdowns in the late 1960s. However, the past two decades have witnessed a renaissance in psychedelic science, marked by rigorous clinical trials and neuroscientific advances [5]. In the 1980s, advancements in molecular pharmacology allowed researchers to understand better how psychedelics mediated their effects on the brain. While it had been long understood that psychedelics interacted broadly with serotonin receptors, researchers including Glennon, Titeler and Nichols demonstrated that hallucinogenic potency correlated strongly with affinity for the serotonin-2 (later classified as 5-HT_2_A receptor [6,7].

Recent findings suggest that psychedelics may be useful for disorders marked by rigidity—such as depression, PTSD, and obsessive–compulsive disorder (OCD)—by promoting neuroplasticity and enhancing cognitive and behavioral flexibility [8,9]. Rigidity refers to persistent, inflexible patterns of thought, emotion, or behavior that make it difficult for individuals to adapt to new information, experiences, or perspectives [10]. Cognitive rigidity specifically is defined by difficulty in shifting mental frameworks or considering alternative viewpoints (e.g., rumination in OCD) [11]. Behavioral rigidity is defined by repetitive or compulsive behaviors that feel impossible to break (e.g., avoidance in PTSD) [10]. Recent systematic reviews estimate the point prevalence of eating disorders worldwide at 5.7% for women and 2.2% for men, with rates as high as 13.5% in some populations; prevalence has nearly doubled since the early 2000s, underscoring a growing public health crisis [12]. Given that eating disorders (EDs) like anorexia nervosa (AN) and bulimia nervosa (BN) are similarly characterized by rigid, perseverative patterns of thought and behavior, it is plausible that psychedelics could offer therapeutic benefit.

Although related disorders, AN and BN exhibit different patterns of serotonin (5-HT) pathway alteration as measured by positron emission tomography (PET). Patients with AN demonstrate reduced 5-HT_2_A receptor function whereas patients with BN show increased 5-HT_1_A receptor function [13]. Patients who have recovered from AN demonstrate reduced [18F] altanserin (a 5-HT_2_A ligand) binding relative to control in the mesial temporal (amygdala and hippocampus) and cingulate cortical regions [14]. Individuals who have recovered from BN show persistent serotonergic dysregulation, including elevated 5-HT_1_A receptor binding and reduced 5-HT_2_A receptor availability in frontal and cingulate regions [15].

In contrast to selective serotonin reuptake inhibitors (SSRIs) which increase serotonin levels in the synaptic cleft by blocking reuptake, serotonergic psychedelics act as partial agonists at the 5-HT_2_A receptor. In this way, psychedelics may offer an alternative approach to serotonin signaling modulation. By transiently altering serotonergic neural network dynamics, they may disrupt rigid patterns of thought, emotion, and self-perception—mechanisms thought to underlie the cognitive and affective inflexibility central to AN, BN, and binge eating disorder (BED) [16].

## 1. Eating Disorders Are Characterized by Cognitive Inflexibility

Impaired cognitive-behavioral flexibility is a core feature of the “eating disorder personality type,” which is characterized by perfectionism, obsession, and rigidity [17]. It should be noted that while cognitive inflexibility is common to patients with AN and BN, these syndromes are distinct disorders. For example, AN and BN are distinguished by low versus high novelty seeking, respectively [18]. A limitation of this review, therefore, is the inclusion of heterogeneous pathophysiology under a common psychopharmacology hypothesis.

The propensity for rigid thinking may emerge early in development, as childhood obsessive-compulsive traits were found to be important risk factors in the development of eating disorders in adulthood, and childhood perfectionism was found to be a significant risk factor in the development of both AN and BN [19,20,21].

Animal models of activity-based anorexia (ABA) provide evidence for the link between cognitive inflexbility and restricted eating. In the ABA paradigm, animals are given limited access to food (usually 1–2 h per day) while having unrestricted access to a running wheel. Despite the availability of food during feeding windows, these animals often show paradoxical increases in physical activity and progressive weight loss, ultimately leading to self-starvation.

This model mimics key behavioral and physiological features of AN, including hyperactivity in the context of caloric restriction, reduced food intake despite an energy deficit and compulsive or inflexible behavior patterns [22,23]. In ABA rodent models, impaired reversal learning is observed, consistent with the cognitive inflexibility of human AN. But unlike humans with AN, ABA rodents typically perform normally on set-shifting tasks [24]. This dissociation highlights both the utility and limitations of ABA as a model for the cognitive inflexiblity of AN.

Human subjects with EDs perform poorly on set-shifting tasks, an executive function that reflects the ability to flexibly switch between different mental sets or tasks. ED patients and their unaffected sisters display poor set-shifting ability as compared to healthy controls, as measured by performance on the Wisconsin Card Sorting Task (WCST) [25,26,27]. The observation that unaffected sisters also displayed poor set-shifting ability hinted at a genetic contribution to cognitive inflexibility. Additional lines of evidence to support this finding include the observation that ED patients are more likely to have healthy first-degree relatives displaying elevated perfectionism (Lilenfeld et al., 1998 [28]) as well as a recent genome-wide association study (GWAS) suggesting a genetic basis for performance on the WCST [28,29].

Objective measures in animal models and human set-shifting data map well to clinical descriptions of the cognitive inflexibility associated with eating disorder diagnostic criteria and phenotypes. Some have proposed that the major eating disorders, namely AN, BN, and BED, represent a continuum of varying obsessive–compulsive versus impulsive personality traits. Vitousek and Manke point out that individuals with AN consistently exhibit personality profiles characterized by constriction, conformity, obsessionality and marked rigidity [30]. Patients with AN have significantly higher total scores on the Multidimensional Perfectionism Scale than healthy controls [31]. While patients with BN can exhibit many of these traits, they tend to exhibit greater affective instability and impulsivity [30]. The presence of impulsive traits may increase the risk of a poor treatment outcome and a worse long-term prognosis in BN [32]. These clinically distinct populations are therefore considered separately in the review that follows.

## 2. Cognitive Flexibility Depends on Healthy 5-HT_2_A Signaling

Emerging data suggest the 5-HT_2_A receptor plays an important role in modulating cognitive flexibility, and in turn, stopping ineffective behaviors and adopting new and more adaptive responses to changing circumstances. Cognitive flexibility can be measured through reversal learning tasks, which are designed to detect perseveration of outdated responses interfering with goal achievement [33,34]. Animal studies have shown that 5-HT_2_A receptor antagonism impairs reversal learning. Conversely, other studies have shown that activating these receptors using psychedelic drugs improves the ability to abandon compulsive, repetitive behaviors [35,36].

In humans, reduced 5-HT_2_A receptor availability is associated with poorer response inhibition, reinforcing its importance in adaptive decision-making [37]. In an extensive literature review, Aznar and Hervig [33] propose that optimal 5-HT_2_A signaling—neither too high nor too low—is essential for balancing rigid and flexible behaviors. Although more research is needed, there is broad consensus that serotonergic signaling is critically involved in cognitive and behavioral flexibility [38].

Serotonin regulates eating behavior and traits linked to EDs, including perfectionism, impulsivity and mood-regulation problems [39,40]. Neuroimaging studies show binding abnormalities of both 5-HT_1_A and 5-HT_2_A receptors in acutely ill and recovered eating disorder patients, respectively [41,42]. Specifically, in acutely ill women with AN, a significant (30–70%) increase in 5-HT_1_A binding potential was seen in prefrontal and lateral orbital frontal regions, mesial and lateral temporal lobes, parietal cortex, and dorsal raphe nuclei compared with control women, while 5-HT_2_A receptor binding potential was normal [42]. However, in 10 women with recovered BN, 5-HT_2_A binding potential was reduced relative to control in the left subgenual cingulate, the left parietal cortex, and the right occipital cortex [41]. Other studies in AN have shown reduced 5-HT_2_A receptor binding before and after recovery. 5-HT_2_A neuroimaging studies in BN are less conclusive, though sample sizes are small. A PET study in healthy adults showed that lower cortical 5-HT_2_A receptor availability is associated with higher levels of trait harm avoidance (anticipating negative outcomes and avoiding them), which is associated with behavioral rigidity [43].

Notwithstanding functional imaging findings implicating serotonin signaling, other pathways likely contribute to various EDs. For example, altered dopamine D2/D3 receptor availability in the striatum has been described in individuals with AN and BN [44]. Glutaminergic dysfunction [45] and alterations in reward-related brain areas such as the acetylcholine (ACh) and opioid systems occur in response to binge eating palatable foods [16].

## 3. Therapeutic Serotonin Modulation: Mechanistic Differences Between SSRIs and Psychedelics

Commercially available serotonin modulators offer some interesting real-world clues regarding the therapeutic potential of modulating serotonin in EDs. Serotonin agonists like fenfluramine (and its active metabolite norfenfluramine) have clear anorectic effects [46], while selective serotonin reuptake inhibitors (SSRIs) are often associated with gradual weight gain [47]. These paradoxical findings led researchers to propose that EDs do not derive from serotonin deficiency, but a more complex dysregulation involving receptor subtype sensitivity and impaired feedback regulation (Brewerton [48]). In particular, findings of altered post-synaptic receptor sensitivity in BN and differential serotonergic signaling in AN have suggested that EDs reflect a broader dysfunction in serotonergic tone, likely shaped by chronic stressors such as dieting, binging, and purging (Brewerton [48]).

While these insights laid the groundwork for the use of SSRIs in the treatment of EDs, clinical results have been inconsistent. Fluoxetine is ineffective in low-weight AN patients, but may help prevent relapse after weight has been restored [49,50]. There are several hypotheses for why fluoxetine and other SRIs do not work in low-weight AN. Fluoxetine selectively inhibits the serotonin transporter (SERT) on presynaptic neurons, blocking the recycling of serotonin (5-HT) from the synaptic cleft back into the neuron. More serotonin in the synaptic cleft prolongs activation of postsynaptic 5-HT receptors. However, in AN, neurotransmitter levels are typically depleted in the absence of L-tryptophan and other dietary precursors [48]. Without sufficient central serotonin available, it could be expected that reuptake would be ineffective. But even after weight restoration, results are still mixed for fluoxetine. In a randomized study, fluoxetine protected AN patients from relapse [49]. However, two other studies showed limited to no benefit on top of cognitive behavioral therapy (CBT) in this setting [50,51].

Fluoxetine is FDA-approved for the treatment of BN. In an 8-week dose ranging trial of 20 mg vs. 60 mg vs. placebo, the 60 mg dose reduced both binging and vomiting frequency [52]. In a subsequent study of maintenance therapy, fluoxetine reduced the risk of relapse as compared to placebo [53]. In binge eating disorder (BED), CBT alone was superior to all other interventions, including fluoxetine combined with CBT [54]. Atomoxetine, an SNRI with no direct effect on serotonin (5-HT) transporters or receptors, reduced binge episodes likely by reducing obsessive cravings rather than appetite suppression [55].

Serotonergic psychedelics such as psilocybin and dimethyltryptamine (DMT, a component of ayahuasca) have demonstrated 5-HT_2_A dependent plasticity-promoting effects. After ingestion, psilocybin is dephosphorylated to psilocin, an agonist of the 5-HT_2_A receptor. 5HT_2_A receptors are densely expressed in apical dendrites of layer 5 pyramidal neurons in the medial frontal cortex of primates and rodents [56,57,58]. Hypothesizing that psilocybin modifies the dendritic architecture of the medial frontal cortex, Shao and colleagues showed that a single dose of psilocybin in mice increases spine density, spine head width and spine protrusion length [59]. The authors showed that a fraction of the psilocybin-induced spines persisted for at least a month after dosing. Ly and colleagues showed similar results for DMT on rat dendritic spine growth and dendritic arbor complexity [60]. Using ketanserin, a selective 5-HT_2_A antagonist, the authors showed they could abrogate the ability of DMT (and psilocin) to promote spinogenesis.

By inducing neuroplasticity, 5-HT_2_A receptor agonism may open a therapeutic window for modifying entrenched patterns of thought and behavior characteristic of eating disorders. Rodent studies provide direct evidence that psilocybin improves cognitive flexibility. Psilocybin was found to acutely improve performance in set-shifting tasks; this effect was blocked by the co-administration of 5-HT_2_A antagonist kerastatin, but not a 5-HT_2_C antagonist, implicating 5-HT_2_A involvement; however, ketanserin alone also improved flexibility, complicating the idea that 5-HT_2_A activation is solely responsible [61].

A single dose of the psychedelic 2,5-dimethoxy4-iodoamphetamine (DOI), a selective 5-HT_2_A agonist, was found to enhance both brain structural plasticity and cognitive flexibility in mice, with effects persisting well beyond the acute treatment window. Improvements in reversal learning and novel decision strategies were time-dependent and shaped by intervening experiences, suggesting that psychedelics may promote adaptive behavior through delayed, experience-contingent neuroplasticity [62]. Conn and colleagues reported that psilocybin improves reversal learning and weight maintenance in a rodent ABA model [63]. However, these authors reported that both 5-HT_1_A and 5-HT_2_A receptor subtypes differentially drive psilocybin-induced flexible learning. They generated evidence that psilocybin may cause a transient shift in the balance of 5-HT_1_A and 5-HT_2_A receptor mRNA transcripts in the prefrontal cortex.

In human non-ED studies of psilocybin, there has been preliminary evidence of cognitive flexibility enhancement, though results are mixed and vary by dose, timing, and population. Microdosing as a strategy for inducing cognitive flexibility appears to be ineffective [64]. Psilocybin produced sustained positive changes in cognitive flexibility in patients with major depressive disorder and increased self-reported flexibility in healthy subjects, particularly among those reporting stronger mystical experiences [65,66]. Another study looked at the differential effects of psilocybin on divergent thinking vs. convergent thinking [67]. The authors reported that while convergent and certain aspects of divergent thinking decreased during the acute psychedelic state, one aspect of divergent thinking was shown to increase 7 days later vs. placebo. The authors hypothesize, “…it could be suggested that the ability of psilocybin to acutely decrease [convergent thinking] and increase spontaneous [divergent thinking], while subacutely enhancing more goal-directed [divergent thinking], could aid in the therapeutic process by opening up a window of opportunity where therapeutic interventions could prove more effective. Namely, while under the influence of a psychedelic, rigid thought content could be decreased, while unguided, spontaneous thoughts may give rise to new insights and perspectives of previous events and current problems.”

## 4. Clinical Evidence for Psilocybin in EDs

The first case study of psilocybin was reported from France in 1959 [68]. This review will focus on the modern era. Table 1 lists all recent psilocybin studies with a Clinicaltrials.gov entry. All remain unpublished, though interim results for several were summarized by investigators in remarks appearing in [69].

Peck and colleagues studied psilocybin in open-label feasibility study that enrolled 10 adult female participants meeting DSM-5 criteria for AN or partial remission AN [70]. The study was designed to characterize safety, tolerability and exploratory efficacy of a single 25 mg dose of psilocybin in conjunction with psychological support. Few details on the type and intensity of the psychological support were provided. The most commonly reported adverse events were headache (80%), fatigue (70%), and nausea (30%). There was a 20% incidence of “feeling abnormal,” migraine, dizziness, and illusion. Two cases of hypoglycemia were also reported. AEs were mild and transient.

Patients were assessed with the Eating Disorder Examination (EDE) questionnaire at study entry and at 1 and 3 months. Using descriptive statistics uncontrolled for multiple comparisons, the authors reported significant differences on the weight concerns subscale at all timepoints. Shape concerns decreased at 1 month follow-up and were no longer significant at 3 months. Four participants (40%) demonstrated global EDE scores that decreased to within 1 s.d. of community norms, which the authors interpreted as clinically significant. At 3 months, 90% “felt more optimistic regarding [their] life endeavors,” 80% felt that “psilocybin dosing was one of the top five most meaningful experiences of [my] life,” and 70% felt that “the overall quality of [my] life has improved” [70]. The authors conclude by calling for larger, controlled trials of psilocybin in AN. They question whether psilocybin therapy may be more effective for certain subsets of AN. They note that it will be important to clarify the dose of psilocybin administered, the optimal number of doses and the role of adjunctive treatments to maximize patient benefit.

Spriggs and colleagues recently conducted a study of psilocybin in AN at Imperial College London (NCT04505189). The study design was informed by focus group input from 11 patients suffering from AN. The study opened in May of 2021 and concluded in June of 2024. Eligible patients were females ages 21 to 65, with a diagnosis of AN present >3 years and for which current or past treatments had not been successful. Patients consented to 8 study visits, including 3 psilocybin dosing sessions at varying doses (maximum dose of 25 mg). Study interventions included 2 MRI scans, up to 5 EEGs and a range of psychological questionnaires and interviews. Follow-up persisted for 12 months following the final study visit. The investigators stated they planned to use the results to shape an eventual randomized control trial. As of this writing, results were still forthcoming, though it was reported that “there was generally good tolerability and promising efficacy data at the end of the 6-week trial period and at 3-month follow-up—as based on the Eating Disorder Examination (EDE), global scores” [69].

Gukasyan and colleagues also recently conducted a study of psilocybin in AN at Johns Hopkins University (NCT04052568). The study opened in August of 2021 and concluded in April of 2023. This was an open-label pilot study of up to 4 doses of psilocybin (with doses between 20 and 30 mg) in combination with psychotherapeutic support. Eligible patients were at least three years from diagnosis and had had at least one previous attempt at treatment. “Preliminary findings from 19 participants showed medium to large effect sizes for improvements in quality of life, ED symptom severity, and mood at one-month post treatment,” with a manuscript in preparation [69]. Adverse events included mild nausea and headache. One serious adverse event occurred in a patient with symptomatic bradycardia that required inpatient monitoring. Another patient experienced syncope during a treatment session [69].

In a study of body dysmorphic disorder (BDD), Zhu and colleagues studied 8 adults who received a single 25 mg dose of psilocybin [71]. While not an ED, BDD is included in this review because patients with BDD also show impaired attentional set-shifting [72], and BDD often co-occurs with EDs [73]. Moreover, due to the paucity of reported clinical data for psilocybin, the results of this study may provide interesting secondary support for the therapeutic hypothesis. By functional MRI, the authors found that psilocybin significantly increased functional connectivity in several brain networks. These included within-network connectivity in the Executive Control Network (ECN)—particularly between the dorsolateral prefrontal cortex (dlPFC) and the superior parietal lobule (SPL)—as well as increased connectivity between the ECN and two other key networks: the Default Mode Network (DMN) and the Salience Network (SN). Clinical efficacy was assessed in 12 subjects (inclusive of the 8 from the fMRI study) using the BDD-YBOCS clinician-rated scale [74,75]. BDD-YBOCS, showed a significant reduction over the 12-week period (*p* < 0.001). Improvements were evident by week 1 and sustained through week 12. 7 of 12 participants (58%) met response criteria, defined as a ≥ 30% reduction in BDD-YBOCS score.

## 5. Clinical Evidence for Ayahuasca in EDs

Ayahuasca refers to plant-based brew that originated from indigenous peoples of the Amazon. Its components include the *Banisteriopsis caapi* vine and leaves from the *Psychotria viridis* bush. *B. caapi* contains the monoamine oxidase inhibitors (MAOIs) harmine, harmaline, and tetrahydroharmine, while *P. viridis* contains the psychoactive alkaloid DMT. It is thought that *B. caapi*’s inhibitory MAO activity potentiates the effects of DMT by protecting it from deamination in the GI tract so that it can become orally available [76]. People who consume DMT report intense visual and cognitive experiences, often with mystical and transcendent themes [77].

Ayahuasca is traditionally given under the care of a ceremony leader. Since traditional ritualistic use includes both dietary restriction and purging—behaviors common to ED pathology—there were questions about its safety in these diseases [78]. In a safety feasibility study, Lafrance and colleagues recruited research subject diagnosed with an ED who had sought out ayahuasca on their own [79]. 14 women and 2 men agreed to recorded phone interviews in which they reported on their experiences with ayahuasca. A 3-member team of researchers adjudicated the call transcripts, looking out for themes that unified the psychedelic experience reports. 11 of 16 research subjects reported reductions in ED thoughts and symptoms, of varying significance. The authors report in part, “In many instances, participants reported enduring positive effects (i.e., lasting months or years), ranging from diminished or more easily managed symptoms to full and sustained remission. They also described attaining new insights about the root causes of their illness, experiencing greater self-love and acceptance, as well as an increase in their capacity to experience and regulate painful emotions.”

In a follow up study, 13 participants with a diagnosed ED also reported on their experiences from an ayahuasca ceremony [80]. The authors reduced these interviews to five themes common among the participants: (1) ayahuasca is an effective form of healing from an ED, (2) ayahuasca allows for deep healing, (3) ayahuasca allows for the processing of intense emotions and/or memories, (4) ayahuasca allows for the embodiment of love, self-love and self-care and (5) ayahuasca provides a spiritual component to healing/recovery. In their discussion, the study authors do not envision ayahuasca replacing conventional treatment. Rather they call for its further study as an adjunct to standard psychotherapy.

## 6. Clinical Evidence for Other Psychedelics in EDs

MDMA is a pharmacologically promiscuous promoter of the serotonin (5-HT), dopamine (DA), and norepinephrine (NE) pathways. It was studied in a randomized Phase 3 trial of 90 subjects with post-traumatic stress disorder (PTSD) (NCT03537014) [81]. The study incorporated the Eating Attitudes Test 26 (EAT-26) as “a pre-specified exploratory measure” because the authors hypothesized that (1) a subset of patients would have high baseline scores on the EAT-26/eating disorder symptoms despite not being underweight or actively purging, and (2) MDMA might reduce EAT-26 scores, especially in women [82,83]. Their hypotheses were based on a deep literature showing that PTSD and related symptoms are commonly associated with eating disorders [84]. In 82 subjects who completed both baseline and follow-up EAT-26 assessments, a significant reduction was observed in the MDMA group vs. placebo; however, these changes were not considered clinically meaningful.

## 7. Other Non-Psychedelic Therapies for BN and BED Modulate Serotonin Signaling

As discussed above, fluoxetine is the only FDA-approved drug treatment for BN [52]. Fluoxetine is recommended in the American Psychiatric Association Practice (APA) Guidelines for BN [85], “require[s] more research before definitive recommendations can be made,” in Canadian practice guidelines [86], and is not recommended by NICE (UK) [87]. Serotoninergic medicines with weaker levels of evidence that are not standards of care in BN include ondansetron, a peripherally active antagonist of the serotonin receptor 5-HT_3_, which improved binge-eating and vomiting in randomized, double-blind, placebo-controlled study [88]. And topiramate, a non-serotonergic multitargeted anti-seizure medication, was associated with improvements in binge and purge symptoms in a randomized trial [89].

In BED, duloxetine, a serotonin-norepinephrine reuptake inhibitor (SNRI), showed modest effects on reducing binge eating, but body mass index and measures of eating pathology, depression, and anxiety did not differ between the two groups [90]. Atomoxetine, a norepinephrine reuptake inhibitor (NRI) showed small but significant reductions in binge eating, weight and BMI after 10 weeks [55]. Lisdexamfetamine (an amphetamine-like stimulant) is FDA-approved for the treatment of moderate-to-severe BED [91]. APA guidelines “suggest” that “adults with binge-eating disorder who prefer medication or have not responded to psychotherapy alone be treated with either an antidepressant medication or lisdexamfetamine” [85]. Canadian and NICE do not recommend any of the above medical therapies for BED [86,87].

There are no known effective serotonergic non-psychedelic medications for AN. Lithium may be effective but is generally contraindicated in dehydrated patients due to increased risk of renal toxicity [92]. Olanzapine can be helpful in the outpatient setting for weight restoration and psychological symptom improvement, though benefits could not be discerned in the setting of intensive residential treatment [93,94,95]. Zinc is often depleted in the setting of semi-starvation, which may further decrease appetite [96]. A number of studies support the use of zinc therapy as an adjunct to other interventions, especially in light of its benign safety profile [97].

## 8. Psychedelics Pose Additional Challenges for Clinical Research

Since most psychedelics are DEA Schedule 1 drugs, extra complexity surrounds the opening of a trial. In addition to the standard clinical research procedures of filing an investigational new drug (IND) application with the FDA and submitting for institutional review board (IRB) approval, investigators must register with the DEA under Form DEA-225. Comparable requirements exist internationally. In Canada, researchers must obtain a Section 56 exemption from Health Canada under the Controlled Drugs and Substances Act, in addition to clinical trial authorization and research ethics board approval. In the UK, investigators must secure a Schedule 1 research license from the Home Office and obtain Medicines and Healthcare products Regulatory Agency (MHRA) approval. In the European Union, researchers must apply for authorization through their national competent authorities under the EU Clinical Trials Regulation (CTR) and may also require special licenses for handling Schedule I/Category 1 substances from national ministries of health or justice.

Research sites must demonstrate they have secure storage procedures, diversion prevention practices and adequate recordkeeping systems. The DEA typically conducts a site inspection, and takes several months before issuing a Schedule I research license. Health Canada and the UK Home Office conduct similar inspections, while in the EU, inspection responsibilities fall to national competent authorities. Finally, acquiring the drug form an authorized source is also complicated. Major academic institutions including Johns Hopkins, NYU, Yale, and Imperial College London have established dedicated psychedelic research centers capable of managing this complexity. There are over 100 clinical trials exploring psychedelics on ClinicalTrials.gov.

Psychedelics may cause intense hallucinogenic effects and thus must be given in an observed setting. In most cases CBT is an ongoing parallel intervention whose methods and impact are hard to capture in the context of a clinical protocol focused on the psychedelic. In other words, it should be assumed that study participants have received CBT prior to study entry and receive it again following study participation. It is therefore difficult to standardize off-study CBT or to capture how this parallel intervention interacts with the investigational therapy. In addition, on study psychological support is never well defined, and no evidence validating how psychological support should be administered is provided. Large randomized designs that make explicit recommendations regarding on-study psychological support and peri-study CBT can minimize some but not all of these confounders.

Placebo control designs are challenging, as study subjects and investigators are functionally unblinded by the psychotropic effects of the investigational agent [98]. Although psychedelic-esque analogs with reduced hallucinogenic potential are being explored [99], it remains to be seen whether they are as effective, since it is unknown whether the spiritual, transcendent and existential effects of traditional psychedelic agents are essential to their putative therapeutic effects [66,100]. When study participants describe psilocybin as “one of the top five most meaningful experiences of [my] life” or ayahuasca as giving them “new insights about the root causes of their illness, experi-encing greater self-love and acceptance…” it is hard to know whether these qualitative insights about the self are essential to the therapeutic hypothesis or just interesting side effects of an intervention that introduces neuroplasticity [70,79]. There is much debate around this topic, but it is beyond the scope of this chapter.

The risks associated with psychedelics receive heightened regulatory scrutiny. In a recent high profile example, a non-profit called The Multidisciplinary Association for Psychedelic Studies (MAPS) set up a for profit entity to secure FDA approval for MDMA in the setting of PTSD [101]. Positive headline results from clinical trials garnered much public interest. But an FDA public advisory meeting was held where panelists raised concerns about study participants who may have experienced severe mental distress that was not accurately captured, including an incident of suicidal ideation [102]. Additional concerns included trial methodology (functional unblinding), therapist bias, limited participant diversity, and ethical lapses. Panelists also highlighted gaps in long-term safety data, questions about cardiovascular risks and potential misuse, and uncertainty about whether the observed benefits were attributable to MDMA itself versus the intensive psychotherapy provided [103,104,105]. The FDA subsequently declined the application, requesting that additional trials be conducted.

## 9. Reasons for Optimism

It appears that most psychedelics do not result in the long-term, compulsive use patterns associated with addiction and are generally safe [106]. Halpern and Pope [107] reviewed nine studies assessing whether LSD and other hallucinogens cause lasting neuropsychological deficits. While some reports historically warned of severe personality changes or cognitive decline in frequent users, the available evidence suggested that any residual effects were modest and often confounded by other factors, such as prior substance use or psychiatric conditions. The authors acknowledged that larger, controlled studies of chronic users were needed [107]. More recently, ref. [108] conducted a systematic review of 34 contemporary experimental studies examining the long-term effects of classic psychedelics, primarily psilocybin, in human participants. The review found evidence of enduring positive changes in mood and well-being, and reductions in depression and anxiety, but rare long-term adverse effects. Elsewhere in the literature, the most serious reported issue associated with psychedelics is Hallucinogen Persisting Perception Disorder (HPPD), a transient visual disturbance including afterimages or visual snow; Type II HPPD, which involves persistent visual disturbances that can interfere with daily life, occurs with an estimated incidence of ~1 in 50,000 [109].

In 2019 the United States Food and Drug Administration (FDA) granted marketing approval to the S-enantiomer of ketamine (also known as esketamine) for patients with treatment-resistant depression [110]. Ketamine is often thought of as a “psychedelic adjacent” medicine because of its dissociative and hallucinogenic side effects. As of January 2025, esketamine had been administered to more than 140,000 patients worldwide [111]. To enable sales, the sponsor had to educate prescribers on billing/reimbursement, patient monitoring, and how to navigate FDA’s Risk Evaluation and Mitigation Strategy (REMS) program. Despite slow initial uptake, this medication is now considered a commercial success, which has likely increased R&D investment in this and related classes of medicines [112]. Clinical studies exploring psychedelics in indications outside of eating disorders are showing preliminary signs of activity [113]. There is encouraging preclinical evidence in neurological disorders including Alzheimer’s dementia [114] and Parkinson’s disease [115], and clinical evidence emerging in non-eating disorder psychiatric illnesses, namely depression [116], OCD [117] and addiction [118]. As academic and commercial interest in this drug class grows, further investigation into eating disorder applications may attract more resources. Additionally, broad research into psychedelics across neurologic and psychiatric indications will expand the overall safety database, benefiting future studies in all potential uses.

## 10. Conclusions

In sum, a growing body of preclinical and clinical research suggests that serotonergic psychedelics acting on the 5-HT_2_A receptor may offer a novel approach to treating eating disorders by targeting the cognitive and behavioral rigidity that underlies their pathophysiology. Unlike SSRIs, which indiscriminately increase synaptic serotonin, psychedelics appear to transiently enhance neuroplasticity, support psychological insight, and promote flexible cognitive reappraisal, which are mechanisms that are particularly relevant in the treatment of AN, BN, and BED. Early human trials, though small and largely uncontrolled, have demonstrated feasibility and signal potential efficacy, while animal studies provide mechanistic insight into their capacity to modify entrenched behavioral patterns. Yet despite these promising findings, significant challenges remain. Regulatory barriers, methodological limitations, and ethical concerns continue to complicate research, and it remains unclear which patients are most likely to benefit, what the ideal therapeutic setting should entail, or how long-lasting the benefits may be. Nevertheless, the renewed scientific interest in psychedelic-assisted therapies, and the early signs of efficacy in intractable conditions, warrants cautious optimism and continued exploration. As the field advances, it will be critical to conduct well-powered, controlled trials that integrate neuroscientific, psychological, and pharmacological perspectives to fully elucidate the therapeutic potential of psychedelics in the context of eating disorders.

## Figures and Tables

**Table 1:** Psilocybin Clinical Studies in EDs.

Trial	Year Opened	Condition	Sample Size (N)	Sites	Contact	Status
NCT04052568	2019	AN	22	Johns Hopkins University	Roland Griffiths, PhD	Completed
NCT04505189	2021	AN	21	Imperial College London	Meg J Spriggs, PhD	Completed
NCT05035927	2022	BED	5	University of Florida Health System	Jennifer L Miller, MD	Completed
NCT05481736	2022	AN	32	Altman Clinical and Translational Research Institute; Sheppard Pratt Health System; Dell Medical School; Tallaght University Hospital; Kings College London, Institute of Psychiatry, Psychology and Neurology	Compass Theraputics	Completed

## Data Availability

Not applicable.
